# Lessons learned in allergy and immunology training: a survey analysis

**DOI:** 10.1186/s13223-022-00649-3

**Published:** 2022-01-31

**Authors:** Chloe Cyr, Michael Cyr, Jaclyn Quirt, Lori Connors

**Affiliations:** 1 Medical Sciences Undergraduate Program, Dalhousie University, 5657 Spring Garden Rd, Suite 503, Halifax, Canada; 2 Medicine, McMaster University, Hamilton, Canada; 3 Medicine, Dalhousie University, Halifax, Canada

**Keywords:** Allergy and immunology, Transition to practice, Competence by Design, Traditional time-based training, Preparedness

## Abstract

**Background:**

There is currently little Canadian data to assess how well traditional time-based residency training programs have prepared residents for careers in Clinical Immunology and Allergy (CIA). This study aims to identify the perceived preparedness of residents in various areas of practice upon the completion of a Canadian CIA residency training program.

**Methods:**

In the summer of 2020, an electronic survey was sent to 2018 and 2019 graduates of Canadian CIA training programs by the Canadian Society of Allergy and Clinical Immunology (CSACI).

**Results:**

Former residents felt well prepared in most Medical Expert areas. Residents felt less prepared for the intrinsic roles of Leader, Communicator, Collaborator, Health Advocate, Scholar, and Professional. The majority of the intrinsic competencies were learned through mentorship and on the job after finishing training.

**Conclusions:**

Upon completion of training, Canadian CIA residents felt well prepared for many competencies, particularly in Medical Expert areas. Training programs may wish to focus on various intrinsic competencies in order to better prepare residents for transition to practice. Academic half-day was not identified as a primary learning centre for intrinsic competencies, suggesting that new teaching strategies may be required.

**Supplementary Information:**

The online version contains supplementary material available at 10.1186/s13223-022-00649-3.

## Background

Clinical Immunology and Allergy (CIA) is a Royal College subspecialty. Individuals can enter a residency in CIA after completing a residency in either Internal Medicine or Pediatrics. There is currently little Canadian data to assess how well traditional time-based training programs have prepared residents for careers in CIA. It is possible that some CanMEDS competencies have not been adequately addressed during training. The CanMEDS framework includes the roles Medical Expert, Communicator, Collaborator, Leader, Health Advocate, Scholar, and Professional [1]. Achieving proficiency in all of these competencies is necessary for residents to successfully transition to practice as independent physicians.

It is anticipated that most residents have adequate training in the Medical Expert CanMEDS role, which is centred on applying medical knowledge and clinical skills [1]. However, it is less clear if residency programs are training residents as well in the intrinsic CanMEDS roles—Communicator, Collaborator, Leader, Health Advocate, Scholar, and Professional.

The Communicator and Collaborator roles involve communicating and working effectively with everyone involved in a patient’s care [1]. The Leader role includes the integration of clinical and administrative responsibilities, whereas the Health Advocate role focuses on improving patient health within the community, and the Professional role emphasizes the importance of doing so ethically [1]. Finally, the Scholar role involves life-long learning [1].

There is concern that some traditional programs do not adequately prepare residents for complete transition to practice. A previous study has shown that American surgical residents feel inadequately trained for the intrinsic roles in areas such as finances, negotiation of contracts, grant application, billing and reimbursement, physician wellness, and running a practice [2]. Most Program Directors in this department also agreed that current residents were under-trained in business and practice management [3].

One current strategy to address teaching intrinsic competencies is through didactic Academic Half-Day teaching. Many Canadian CIA programs participate in a nation-wide Distributed Academic Half-Day, while other programs run their own Academic Half-Day curriculum. Residents may also learn intrinsic competencies through mentorship, community-based rotations, and hospital-based rotations, amongst other sources.

In an attempt to better address the needs of future physicians, the Royal College has undertaken a major change in residency training with the implementation of Competence By Design (CBD). All Royal College training programs are transitioning to CBD, with CIA projected to transition for the 2021–2022 academic year. Some of the key goals of CBD are to “identify the competencies needed at all stages of training and practice” and to “adjust learning to individual needs and abilities.” [4].

In the CBD model, stages of competency are outlined, one of which is transition to practice. The entrustable professional activities (EPAs) identified in this stage include management of complex patients, as well as managing a practice [5]. It is expected that this focus on transition to practice will help residents feel prepared for careers in CIA.

This study aims to identify the perceived preparedness of residents in various areas of practice upon the completion of a traditional Canadian CIA training program. The feedback from recent graduates should help identify the strengths, weaknesses, and possible gaps in the current CIA training programs prior to implementation of CBD, and could inform Program Directors on how best to adapt their programs to meet the needs of future trainees.

## Methods

A short electronic survey was distributed through Opinio. It was anonymous, 15 questions long, and involved checklist, multiple choice, and open-ended questions (Additional file 1: Appendix A). The survey was sent to 2018 and 2019 graduates of Canadian CIA residency programs distributed by the Canadian Society of Allergy and Clinical Immunology (CSACI). Questions were designed to assess how well prepared physicians felt for various areas of practice and included questions pertaining to the CanMEDS roles. Respondents were able to comment on specific weaknesses and knowledge gaps, and were asked to identify where they learned specific competencies. Program Directors were encouraged to share the survey with their former residents and several reminder emails were sent out in order to maximize the response rate. The survey was open for approximately 1 month and respondents were only able to answer once.

## Results

There were 18 respondents to the survey out of a possible 45 respondents (40% response rate). Two thirds of the respondents completed an Adult CIA program and one third completed a Pediatric CIA program. Most respondents (55.6%) had 3–5 residents in their training program in their final year, 38.9% had 1–2 residents, and 5.6% had more than 5. Most respondents (77.8%) completed their training in 2019 and 22.2% completed their training in 2018. Of the respondents, 72.2% reported their gender to be female and 27.8% reported their gender to be male.

The data showed that 77.8% of respondents currently practice in a community-based clinic, 27.8% practice in a community hospital, 33.3% practice in an academic centre, 11.1% are continuing training, and 11.1% are practicing in another environment (ex. private clinic). Half of the respondents reported being in programs that participated in the Distributed Academic Half-Day program, 44.4% did not, and 5.6% were unsure.

Note that if respondents indicated that they did not treat a condition listed under the Medical Expert role (ex. treating autoinflammatory disease), or did not require a specific intrinsic competency in their practice (ex. setting up a research lab), they were excluded from data surrounding that competency.

### Medical expert

**Fig. 1:**
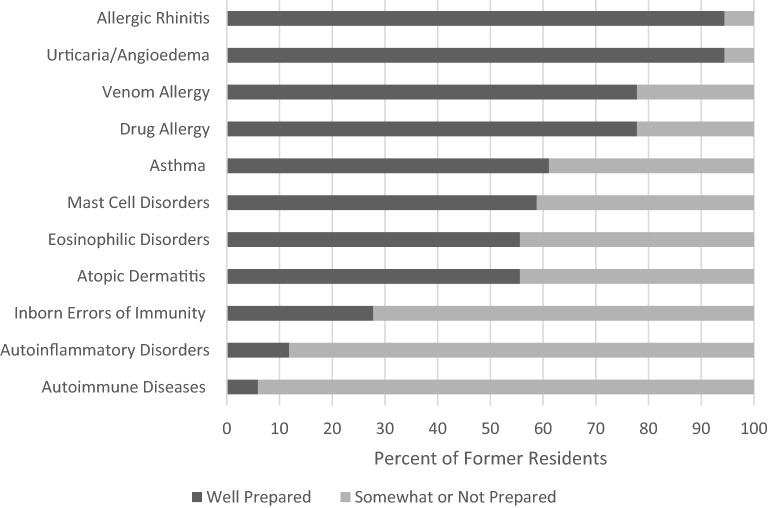
Perceived preparedness of 2018 and 2019 Canadian CIA graduates for various aspects of the Medical Expert role

Most former residents felt well prepared to treat allergic rhinitis (94.4%), urticaria/angioedema (94.4%), venom allergies (77.8%), drug allergies (77.8%), asthma (61.1%), mast cell disorders (58.8%), eosinophilic disorders (55.6%), and atropic dermatitis (55.6%). Most graduates felt somewhat or not prepared to treat autoimmune diseases (94.1%), autoinflammatory disorders (88.2%), and inborn errors of immunity (72.2%) (Fig. 1).

### Intrinsic competencies

**Fig. 2:**
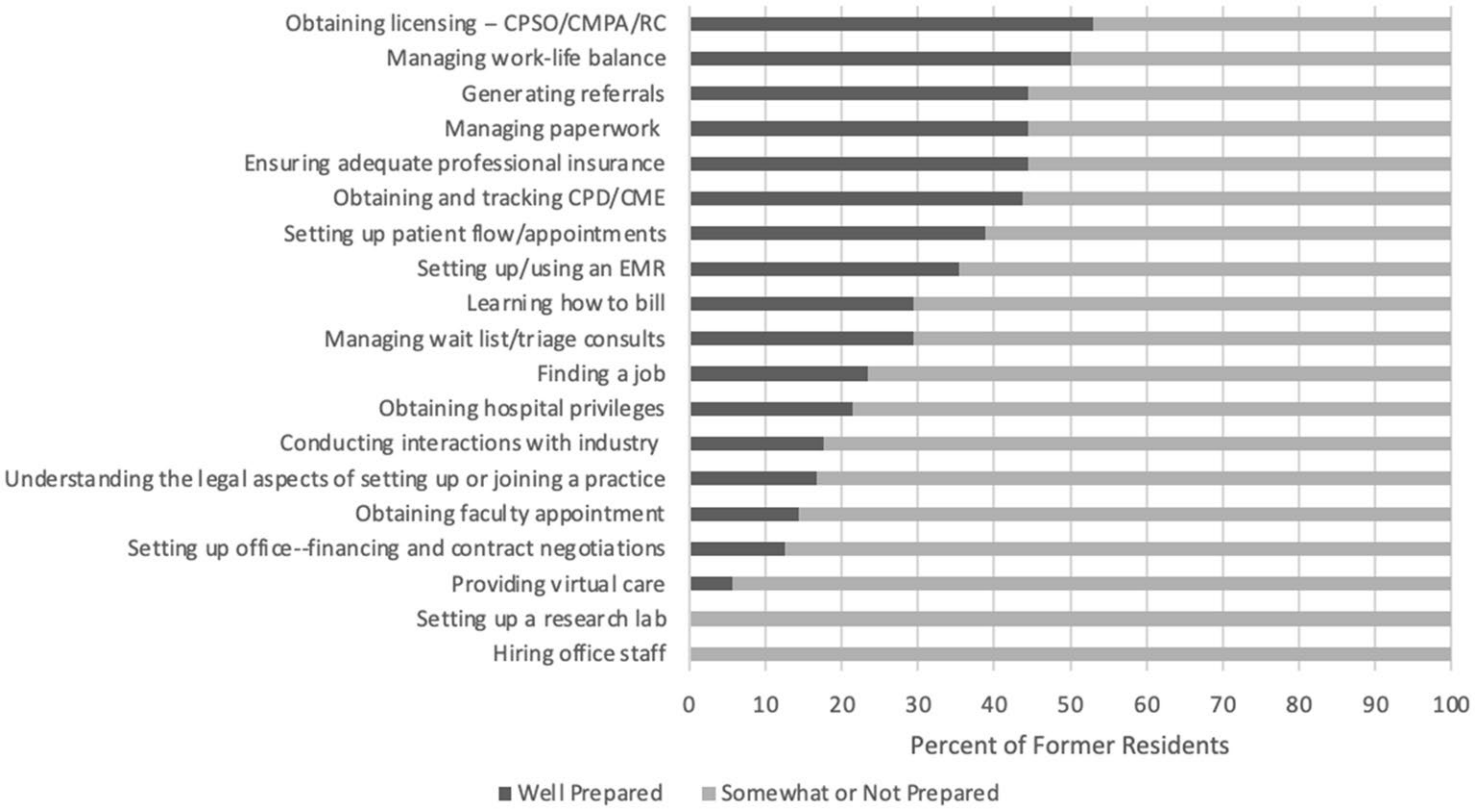
Perceived preparedness of 2018 and 2019 Canadian CIA graduates for various aspects of intrinsic roles

Most former residents felt well prepared to obtain licensing (52.9%). Former residents felt somewhat or not prepared for the remaining competencies pertaining to establishing and managing practice, such as hiring office staff, setting up a research lab, and providing virtual care. Seven former residents did not set up a research lab, five did not hire office staff, four did not obtain hospital privileges, and four did not obtain faculty appointment (Fig. 2).

In a select-all-that-apply question, respondents indicated where they learned specific intrinsic competencies. Former residents indicated that they learned 13 competencies primarily on the job after finishing the program, 5 procedures primarily or equally through mentorship, and 1 through another source. Community rotations, hospital rotations, and academic half-day were not indicated as primary learning centres. However, residents indicated that community rotations were helpful in a written response question. Former residents indicated being surprised about “the effort and time needed to generate a strong referral base” and suggested program teaching have a greater focus on the administrative aspects of practice in order to improve training.

## Discussion

Our study showed that Canadian CIA recent graduates felt well prepared for many Medical Expert competencies. Areas where residents felt particularly well prepared (over 70% respondents indicating well prepared) included managing allergic rhinitis, urticaria/angioedema, venom allergies, and drug allergies. Areas where residents felt less prepared (over 70% somewhat or not prepared) included managing autoinflammatory disorders, autoimmune diseases, and inborn errors of immunity. This suggests that the Immunology component of CIA programs may need to be amplified, which may be centre dependent.

Overall, residents felt less prepared for intrinsic competencies. No intrinsic competencies had over 70% of respondents feeling well prepared. Areas where residents felt the most unprepared (over 85% somewhat or not prepared) included hiring office staff, setting up a research lab, providing virtual care, setting up office, and obtaining faculty appointment. Programs may wish to provide learning experiences that better meet these competencies, such as an office management rotation, a project with a non-for-profit organization, or more community rotations.

Former residents stated that they learned the majority of the intrinsic competencies on the job after finishing training and through mentorship. Residents indicated that they were least likely to learn intrinsic competencies through Academic Half-Day, indicating didactic sessions may not be the best way to teach these competencies. Allowing residents to practice these skills and integrate them into their own practice style may be more beneficial.

There were some areas that several (3 or more) residents indicated that they did not participate in. These areas included hiring office staff, obtaining hospital privileges, obtaining faculty appointment, and setting up a research lab. However, more residents (61%) set up a research lab than expected. This high percentage may indicate that CIA is a field that lends itself to doing lab research and that the training programs may be accepting residents who are predisposed to doing research and/or are preparing and encouraging them to do research. It may also reflect that residents that went on to do research may have been more likely to answer our survey rather than ones who did not. Further study into which intrinsic roles recent graduates find most useful in the first years of practice may help training programs prioritize which areas to focus on during training.

Limitations to the study include the 40% response rate. One possible contributing factor was that this survey was distributed during the COVID-19 pandemic. During this time, physicians were inundated with many emails and surveys which may have affected their willingness to respond to our survey. As in all survey studies, it is possible that respondents were not reflective of the full group. Another potential limitation was that no data was collected regarding the location of the training programs of the respondents. This was done to ensure the anonymity of the respondents was maintained, as many of the training programs are small.

Future research could look at formulating a Transition to Practice (TTP) rotation, such as has been created by Dalhousie University [6]. It may be beneficial for faculty to participate in more Continuing Professional Development (CPD) activities in relation to intrinsic roles. This participation may lead to increased faculty comfort in these areas and thus a better integration of intrinsic role teaching in various formats. This study also provides a baseline for future research to assess the impact of CBD implementation on preparedness for practice in these areas.

Finally, although this study was specific to CIA we feel the survey format used may also be of benefit in other specialties that are largely outpatient/community focused.

## Conclusions

Current traditional time-based Canadian CIA training programs are preparing residents for most Medical Expert competencies. Conversely, more focus should be put onto teaching intrinsic competencies during training in order to better prepare residents for transition to practice. Specifically, integration of intrinsic competencies could be useful in informal settings such as a TTP rotation.

## Supplementary Information

**Additional file 1.** Appendix.

## Data Availability

All data generated or analysed during this study are included in this published article.

## Abbreviations

CBD: Competence By Design
CIA: Clinical Immunology and Allergy
CPD: Continuing Professional Development
CSACI: Canadian Society of Allergy and Clinical Immunology
EPA: Entrustable Professional Activity
TTP: Transition to practice
