# Supplementary material for: Lessons learned in allergy and immunology training: a survey analysis
Source: Allergy Asthma Clin Immunol. 2022 Jan 31;18:7. doi: 10.1186/s13223-022-00649-3 (PMC8802268; doi:10.1186/s13223-022-00649-3)
Supplement: Supplementary file 1 — Additional file 1. Appendix. [file 13223_2022_649_MOESM1_ESM.docx]

**Appendices**

Appendix A

Transition to Practice: Lessons Learned in Allergy and Immunology Training

Now that you have completed your allergy training, we would like to know how prepared you felt to practice in Allergy / Immunology.

Which program did you complete?

Adult Clinical Immunology and Allergy

Pediatric Clinical Immunology and Allergy

How many residents were there in your training program in your final year?

1-2

3-5

More than 5

When did you complete your training?

2018

2019

What is your gender?

Male

Female

Gender diverse

Prefer not to say

What type of practice are you currently working in? Check all that apply.

Community-based clinic

Community hospital

Academic Centre

Research

Continuing training

Not working

Other

Was your training program part of the Distributed Academic Half-Day program?

Yes

No

Unsure

How well prepared do you feel you were for managing the following clinical areas:

| Clinical Area | Not prepared | Somewhat Prepared | Well prepared | I don’t treat this |
| --- | --- | --- | --- | --- |
| Allergic Rhinitis |  |  |  |  |
| Asthma |  |  |  |  |
| Urticaria/Angioedema |  |  |  |  |
| Atopic dermatitis |  |  |  |  |
| Eosinophilic disorders |  |  |  |  |
| Mast cell disorders |  |  |  |  |
| Drug allergy |  |  |  |  |
| Venom allergy |  |  |  |  |
| Inborn errors of immunity |  |  |  |  |
| Autoimmune diseases |  |  |  |  |
| Autoinflammatory disorders |  |  |  |  |

If there is any other clinical area that is pertinent to you, please insert and describe your readiness for it here.

How well prepared do you feel you were for the following procedures:

| Procedure | Not prepared | Somewhat Prepared | Well prepared | I don’t do this procedure |
| --- | --- | --- | --- | --- |
| Setting up office – financing and contract negotiations |  |  |  |  |
| Finding a job |  |  |  |  |
| Hiring office staff |  |  |  |  |
| Obtaining hospital privileges |  |  |  |  |
| Obtaining faculty appointment |  |  |  |  |
| Setting up a research lab |  |  |  |  |
| Obtaining licensing – CPSO/CMPA/RC |  |  |  |  |
| Obtaining and tracking CPD/CME |  |  |  |  |
| Understanding the legal aspects of setting up or joining a practice |  |  |  |  |
| Setting up / using an EMR |  |  |  |  |
| Setting up patient flow/ appointments |  |  |  |  |
| Managing wait list/ triage consults |  |  |  |  |
| Learning how to bill |  |  |  |  |
| Conducting interactions with industry |  |  |  |  |
| Managing paperwork |  |  |  |  |
| Managing work-life balance |  |  |  |  |
| Ensuring adequate professional insurance |  |  |  |  |
| Providing virtual care |  |  |  |  |
| Generating Referrals |  |  |  |  |

If there is any other procedure that is pertinent to you, please insert and describe your readiness for it here.

Where did you learn each procedure? Check all that apply.

| Procedure | Mentorship | Hospital rotations | Community Rotations | Academic half-day | On the job after finishing the program | Other |
| --- | --- | --- | --- | --- | --- | --- |
| Setting up office – financing and contract negotiations |  |  |  |  |  |  |
| Finding a job |  |  |  |  |  |  |
| Hiring office staff |  |  |  |  |  |  |
| Obtaining hospital privileges |  |  |  |  |  |  |
| Obtaining faculty appointment |  |  |  |  |  |  |
| Setting up a research lab |  |  |  |  |  |  |
| Obtaining licensing – CPSO/CMPA/RC |  |  |  |  |  |  |
| Obtaining and tracking CPD/CME |  |  |  |  |  |  |
| Understanding the legal aspects of setting up or joining a practice |  |  |  |  |  |  |
| Setting up / using an EMR |  |  |  |  |  |  |
| Setting up patient flow/ appointments |  |  |  |  |  |  |
| Managing wait list/ triage consults |  |  |  |  |  |  |
| Learning how to bill |  |  |  |  |  |  |
| Conducting interactions with industry |  |  |  |  |  |  |
| Managing paperwork |  |  |  |  |  |  |
| Managing work-life balance |  |  |  |  |  |  |
| Ensuring adequate professional insurance |  |  |  |  |  |  |
| Providing virtual care |  |  |  |  |  |  |
| Generating Referrals |  |  |  |  |  |  |

If there is any other procedure that is pertinent to you, please insert and describe where you learned it here.

Is there anything during your training that you felt particularly helped prepare you for practice?

Is there anything that surprised you about starting practice?

Is there anything that you feel could have improved your training?
